# A transdisciplinary approach to snakebite envenoming

**DOI:** 10.1016/j.toxcx.2021.100088

**Published:** 2021-12-22

**Authors:** Rafael Ruiz de Castañeda, Isabelle Bolon, José María Gutiérrez

**Affiliations:** a Division of Tropical and Humanitarian Medicine & Institute of Global Health, Department of Community Health and Medicine, Faculty of Medicine, University of Geneva, Geneva, Switzerland; b Instituto Clodomiro Picado, Facultad de Microbiología, Universidad de Costa Rica, San José, Costa Rica

Snakebite envenoming (SBE) is one of the most complex of all neglected tropical diseases. First, snakes are a highly diverse group of reptiles, often very hard to study in the wild and at the level of populations, including over 3700 species with more than 650 of them being venomous and with a large intra and inter-specific variation in their venoms and associated SBE victim pathologies. Second, snakes are one of the most culturally symbolic animals and have been represented with different forms and meanings along the history of humankind across the world. Snakes are both feared and venerated, which has diverse implications on the way people interact with these animals and how different cultures perceive, prevent and respond to SBE. Third, although antivenoms have been the mainstay of SBE treatment for over a century, their availability and accessibility, especially in remote rural settings where most SBE cases occur, is still extremely limited. Moreover, the efficacy and safety of many antivenoms available in the market have not yet been fully demonstrated at the preclinical and clinical levels. This scenario is further complicated by the often deficient basic medical facilities and limited capacities to manage SBE in low-resource public health systems. The reality is that preventing, managing, and treating SBE adequately is still challenging in many parts of the world, particularly in rural regions of sub-Saharan Africa, Asia and Latin America, and thousands of men, women and children unnecessarily die or are dramatically disabled every year due to this disease. Fourth, SBE is described as the most neglected of the neglected tropical diseases (NTD) and therefore research on this topic has been poorly funded and undertaken by only a small and specialised research community. SBE has often lacked the necessary interdisciplinarity and inherent opportunities for innovation that result from collaborating across diseases, disciplines, and sectors (Gutiérrez et al., 2015, 2020).

A series of developments in the last decade have contributed to a renewed global awareness on the impact of SBE. After the inclusion of SBE in the official list of NTDs of the World Health Organization (WHO) in 2017, a resolution on this topic was adopted in the 2018 World Health Assembly and then followed by the launch of the 2019–2030 WHO road map on SBE (WHO 2019) and more recently the 2021–2030 WHO road map on NTD (WHO 2020). These developments have generated a positive political momentum and a growing scientific enthusiasm towards working more collaboratively and integratively to better understand and tackle SBE, involving a diverse set of stakeholders. Concepts such as One Health, which has almost become a global health motto in the context of the ongoing COVID19 pandemic, are now central to the WHO SBE and NTD road maps, encouraging collaborations across the human, animal and environmental sectors, and the engagement with and the active participation of the communities suffering from the disease in the research and implementation of public health actions.

This *Toxicon: X* special issue on SBE aligns and supports this transdisciplinary approach and presents the state of the art of the research in this field, bringing together an unprecedented list of 17 articles by an international group of female and male researchers and diverse professionals working for different types of institutions (e.g., academia, international organizations, non-governmental organizations (NGOs), and governmental bodies) and from all SBE endemic regions, including some of the most affected countries in the world. This special issue involves some of the most re-known experts in this subject together with a new generation of SBE researchers, and experts in other diseases that are increasingly attracted by this fascinating domain.

In contrast to some infectious diseases, SBE cannot be eradicated. Snakes play a critical role in ecosystems where they thrive and a healthy co-existence between venomous snakes and people, where the risk of SBE is reduced while the snakes are protected, depends, as suggested by Anita Malhotra et al. in this special issue, on increasing the herpetological knowledge base in SBE endemic areas. Anna Pintor et al. and Andrew Durso et al. contributed with two highly innovative manuscripts on herpetology that highlight the potential of Big data, including both conventional and non-conventional data such as those generated by citizens and crowdsourced via the web, to better understand the diversity and distribution of venomous and non-venomous snakes across the world and inform SBE management from the global to the local levels. Herpetology and SBE have traditionally been data-poor domains and, in the era of artificial intelligence and digital epidemiology, novel forms of data and ways to analyse them with the latest geospatial and modelling tools need to be carefully considered and, where relevant, applied in the design of evidence-based public health interventions to reduce the burden of SBE. On the other hand, Juan José Calvete et al. present a highly original conceptualization of snake venoms viewed from the ecological and evolutionary perspectives, and how they relate to clinical toxinology. Their contribution explores the impact of the ‘omics’ technologies for developing a more holistic view of snake venoms in their ecological and medical contexts.

The study of the impact of SBE has focused on people, with very limited and often anecdotal evidence of the impact of SBE in livestock and companion animals in rural SBE endemic areas. Isabelle Bolon et al. have brought the veterinarian perspective to this special issue with a manuscript that builds on unprecedented nation-wide primary data collected at the community level in Nepal and Cameroon, both SBE hyper-endemic countries. In line with the WHO road map on SBE, their results show a high impact of SBE on livestock animals and subsequent livelihood losses, and reinforce the need to address SBE through a One Health lens. Rabies, another NTD with important applications of the One Health approach, shares multiple aspects with SBE (i.e., both are health emergencies caused by an animal bite, mainly occur in underserved settings, and use animal-derived immunoglobulins as therapeutics among other common features). The article by Terence Scott et al. unites experts on SBE and rabies to discuss common grounds between these two diseases and practical opportunities for synergies and a better and more integrated management.

Social research needs to be better incorporated in the field of SBE and in this special issue we have paid particular attention to this aspect. We have included four manuscripts in this domain, ranging from the review by Bethany Moos et al. on public health community engagement around SBE and other NTDs in East Africa, to the work done by Kieran Barnes et al. Manon Chuat et al. and Romain Duda et al. covering diverse questions for different parts of the world, with emphasis in sub-Saharan Africa. These contributions underscore the importance of understanding the local knowledge and the perceptions on snakes and SBE and their implications for SBE prevention and care. The value of anthropological studies and qualitative research in the field of SBE is evidenced in these works. As illustrated in these contributions, a ‘dialogic’ approach with people from communities suffering a heavy burden of SBE is necessary to fully grasp the complexities of this disease and to generate effective interventions tailored to the local contexts.

The COVID19 pandemic is also having an impact on the efforts to control SBE, and SBE should not anymore be seen in isolation but as part of and affected by the planetary human-driven changes. In this context, Janneke van Oirschot et al. looked at the impact of COVID19 on SBE with first-hand experiences from key informants globally. Moreover, Gabriel Alcoba et al. wrote about SBE in the context of the global humanitarian and migration crisis, and Gerardo Martín et al. provide a critical analysis of what we know and what we need to know about SBE and the ongoing global environmental change. This work shows how the climate change affects in multiple ways the distribution of snakes and the incidence of SBE, in close association with changes in human demographic patterns and the use of land in a context-dependent fashion.

Besides featuring new and exciting areas of work on SBE, overall this special issue aims to highlight the fact that there are relatively simple solutions to make almost immediate and large scale impact on SBE, provided integrated multi-sectorial actions are implemented. Access to safe, effective, and affordable antivenoms and health systems with healthcare professionals trained in the diagnosis and management of SBE are absolutely key. This special issue includes important contributions by Julien Potet et al. on the access to antivenoms in the developing world, and by Muhammad Hamza et al. on the present and future directions of the clinical diagnosis and management of SBE. The long-term effects of SBE, a rather neglected aspect of this disease, is analysed by Janaka de Silva et al. calling for interventions to attend people suffering from these types of consequences. Furthermore, José María Gutierrez et al. present a comparison between the way SBE has been handled in three highly different national contexts (i.e., Costa Rica, Sri Lanka, and Nigeria). This comparison illustrates strengths and weaknesses in these countries and proposes urgent tasks to improve the management of this disease at the national level, involving international cooperation.

We are impressed by the originality and quality of all the contributions, as well as the motivation by all authors and co-authors, and sincerely thank them all. We also thank all the reviewers that have kindly contributed to this special issue with their valuable feedback on the manuscripts. We believe that this special issue has reinforced existing connections between researchers and created new ones, paving the way for the incorporation of colleagues from diverse disciplines, including social sciences, into this field. As the WHO global strategy to prevent and control SBE unfolds, we hope that many of the ideas presented in this special issue will contribute to the design and support of more effective public health interventions aimed at reducing the suffering inflicted by SBE. We also expect to transmit this motivation to our readers, inviting more people from different backgrounds to be part of the global efforts to the fight against SBE.

## Credit author statement

Rafael Ruiz de Castaneda: Conceptualization, Writing – original draft, Writing – review & editing. Isabelle Bolon: Conceptualization, Writing – review & editing. Jose Maria Gutierrez: Conceptualization, Writing – review & editing.

## Ethical statement

This is an Editorial MS and therefore no specific Ethical statement is provided or required in this case.

## Declaration of competing interest

The authors declare that they have no known competing financial interests or personal relationships that could have appeared to influence the work reported in this paper.
